# Radiobiological modeling of interplay between accelerated repopulation and altered fractionation schedules in head and neck cancer

**DOI:** 10.4103/0971-6203.56081

**Published:** 2009

**Authors:** Loredana G. Marcu, Eva Bezak

**Affiliations:** 1 University of Adelaide, School of Chemistry and Physics, North Terrace, 5000 SA, Australia; 2 University of Oradea, Faculty of Science, 1 Universitatii str, Oradea, Romania; 3 Royal Adelaide Hospital, North Terrace, 5000 SA, Australia

**Keywords:** Altered fractionation, Monte Carlo, tumor repopulation

## Abstract

Head and neck cancer represents a challenge for radiation oncologists due to accelerated repopulation of cancer cells during treatment. This study aims to simulate, using Monte Carlo methods, the response of a virtual head and neck tumor to both conventional and altered fractionation schedules in radiotherapy when accelerated repopulation is considered. Although clinical trials are indispensable for evaluation of novel therapeutic techniques, they are time-consuming processes which involve many complex and variable factors for success. Models can overcome some of the limitations encountered by trials as they are able to simulate in less complex environment tumor cell kinetics and dynamics, interaction processes between cells and ionizing radiation and their outcome. Conventional, hyperfractionated and accelerated treatment schedules have been implemented in a previously developed tumor growth model which also incorporates tumor repopulation during treatment. This study focuses on the influence of three main treatment-related parameters, dose per fraction, inter fraction interval and length of treatment gap and gap timing based on RTOG trial data on head and neck cancer, on tumor control. The model has shown that conventionally fractionated radiotherapy is not able to eradicate the stem population of the tumor. Therefore, new techniques such as hyperfractionated/ accelerated radiotherapy schedules should be employed. Furthermore, the correct selection of schedule-related parameters (dose per fraction, time between fractions, treatment gap scheduling) is crucial in overcoming accelerated repopulation. Modeling of treatment regimens and their input parameters can offer better understanding of the radiobiological interactions and also treatment outcome.

## Introduction

While head and neck cancer is not one of the more common cancers, it is among the deadliest. Surgery, radiation and/or chemotherapy have not improved the 50% overall five-year survival of this debilitating disease over the past 30 years. One of the main reasons for the failure of local tumor control in head and neck cancer is accelerated repopulation of cancer cells.[1] Accelerated repopulation is a marked increase in the tumor growth rate (15 to 20 times faster) after the commencement of radiotherapy that becomes measurable, usually three to four weeks, after the start of the treatment. Conventional radiotherapy cannot overcome tumor repopulation. Therefore there is a great need for well defined radiobiology-based treatment schedules. Applying radiobiological principles to combined modality treatment of head and neck cancer, especially the optimization of timing of radiation dose delivery, has shown to enhance tumor response.[2]

The impact of different fractionation schedules are most commonly investigated through clinical trials. While clinical trials are indispensable prerequisites to establish novel therapeutic techniques, they are time-consuming processes which involve several determining factors for success. It is difficult to explore the sensitivity of the outcome to the multitude of input parameters each with its own trial. Consequently, a simpler modeling approach can be employed to describe the radiobiological processes during radiotherapy.

The aim of this work was to implement a modeling approach and simulate the response of a virtual head and neck tumor to radiotherapy using computer-based probabilistic methods (Monte Carlo). The biological mechanisms responsible for tumor repopulation during treatment have been included in the model. Altered fractionation schedules are usually employed in clinics to overcome tumor repopulation. Three main treatment-related parameters (such as: dose per fraction, inter fraction interval and length of treatment gap for the accelerated radiotherapy schedule) and the corresponding clinical values [Table 1] are implemented in the current model and the outcome is analyzed. The ultimate goal was to derive the optimum radiotherapy treatment schedule for head and neck cancer based on the interplay between accelerated repopulation and treatment-related parameters.

**Table 1:** Variation of dose per fraction

*Treatment schedules:*
1.1 Gy twice a day, 5 days a week, over 7 weeks, 77 Gy overall dose 1.2 Gy twice a day, 5 days a week, over 7 weeks, 84 Gy overall dose 2 Gy once a day, 5 days a week, over 7 weeks, 70 Gy overall dose

## Materials and Methods

### Modeling of tumor growth

The growth of a head and neck tumor has been modeled using the Monte Carlo method; the model being presented in details in previously published work.[3] A summary of the simulation process is presented in the following section. The computational model maintains the biological composition of a tumor through the generation of Stem (S), Finitely Proliferating (P) and Non-proliferating cells (N). The modeling process encompasses four main phases: input set up, cell generation and parameterization, timing control, calculation and display of the results. The input module defines and initializes the variables. The main biological variables modeled are: the overall number of cells to be tracked, S:P:N ratio, relative lengths of the phases of the cell cycle, average cell cycle time, cell loss factor, number of generations of proliferative cells and P:N ratio. The cell generation module initiates the creation of new cells starting from a single stem cell simulating, therefore, the biological stage of mitosis. Each newly formed cell is assigned a cell cycle time with a mean value of 33 hours (three times the length of the S phase which for head and neck cancer is 11 hours).[4] The cell cycle time is allocated by randomly sampling from a truncated Gaussian distribution with a standard deviation of 13.7 hours that reflects known biological characteristics. The length of the four phases for each cell is attributed according to the following proportions of the cell cycle: M-7%, G_1_-40%, S-30% and G_2_-23%. To simulate apoptotic death (cell loss due to natural causes), an 85% cell loss[4] is incorporated through sampling from a uniform distribution following cell generation. The flow of cell creation and proliferation is temporally based. The results and display module keeps track of the overall number of cells, number of particular cell types and also cell distribution along the four phases of the cycle.

### Modeling of Radiotherapy

In order to treat this virtual head and neck tumor, both conventional and altered fractionation radiotherapy schedules have been simulated. Conventionally fractionated radiotherapy treatment is given as a daily dose of 2 Gy, five days a week, over seven weeks. Hyperfractionated radiotherapy is characterized by multiple fractions of small doses, given daily (two fractions a day in the present model). The total dose is, usually, the same or moderately increased compared to conventional treatment, while total treatment time is the same. Accelerated radiotherapy is defined as a radiotherapy regimen in which the duration of conventional treatment is reduced by the delivery of two or more treatments on some or all of the treatment days. The treatment can be accelerated by treating six days/week instead of five days/week. Sometimes the total dose is also reduced. The accelerated fractionation regimen often includes a treatment gap (up to a couple of weeks) to allow for normal tissue repair.

The survival fraction of 54%[5] after 2 Gy dose irradiation has been implemented in the simulation, and the linear quadratic model has been used to determine surviving fractions for smaller or larger doses per fraction.

### Accelerated Repopulation Mechanisms

**Figure 1:**
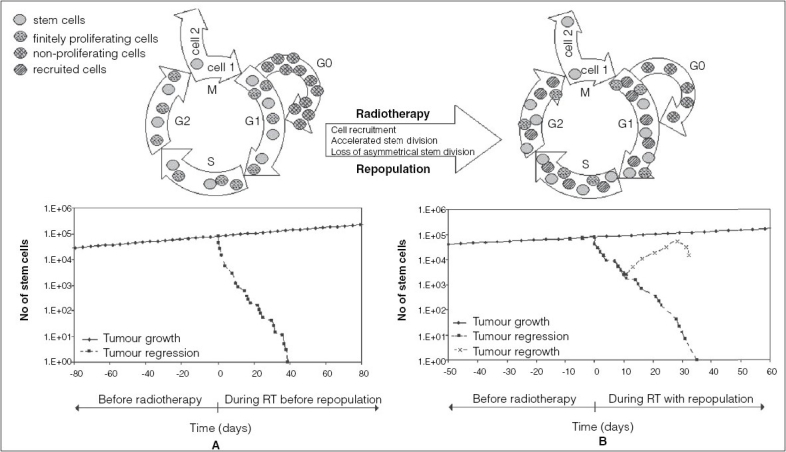
Illustration of unperturbed tumor growth and tumor behavior during radiotherapy with (B) and without (A) Repopulation mechanisms

The main repopulation mechanisms within head and neck tumors that have been implemented in the model are: cell recruitment, accelerated stem cell division and loss in asymmetrical division of stem cells.[6–9] Cell recruitment is the re-entry of the quiescent cells (cells in the G_0_ phase) into the mitotic cycle. Accelerated stem cell division refers to the shortening of the cell cycle time after the start of radiotherapy. Loss in asymmetrical division expresses the change in division pattern of the stem cells, from an asymmetrical division (one stem and another finitely proliferating/nonproliferating cell) to a symmetrical division (two stem cells). The authors have previously published an extensive modeling work with a quantitative assessment of the above defined mechanisms[10]. The present work uses this tumor growth model with the accelerated repopulation mechanisms incorporated, and aims to analyze the effect of various radiotherapy schedules on tumor behavior during treatment. Figure 1 part A is a representation of tumor growth and regression during conventional radiotherapy when no repopulation mechanisms are included in the model. Figure 1 part B illustrates tumor growth, regression and regrowth with repopulation mechanisms implemented. The two cell cycles illustrate the situation before repopulation and during repopulation, respectively, when cell recruitment from G_0_ contributes towards tumor regrowth.

### Modeling of Treatment-related Parameters

One method to overcome accelerated repopulation during treatment of aggressive tumors such as the advanced head and neck cancer is to replace the conventionally fractionated radiation treatment with altered fractionation schedules. The Radiation Therapy Oncology Group (RTOG) phase III randomized study has compared the hyperfractionated radiotherapy schedule with accelerated fractionation in assessing loco-regional tumor control.[11] This study has taken the RTOG trial parameters further, by implementing them in the above described tumor model and including other clinically relevant values to the treatment-related parameters to evaluate tumor control in a comparative manner.

The main schedule-related parameters influencing tumor control that should be taken into account when designing a treatment schedule are: dose per fraction, interfraction interval and the length of treatment gap (for the accelerated radiotherapy). These parameters have been included in the model with their clinical values [Table 1].

## Results

The current work has focused on two main aspects of head and neck cancer radiotherapy: the advantage of altered fractionation over conventionally fractionated radiotherapy and the role of schedule-related parameters and their clinical values on tumor control. The results are:

### Conventional Versus Altered Fractionation Schedules

“The object of treating a tumor by radiotherapy is to damage every single potentially malignant cell to such an extent that it cannot continue to proliferate”.[12] In this model, as in biological settings, the stem (or clonogenic) cells are the ones responsible for tumor growth and repopulation. Therefore, within the model, the focus was on behaviour of stem population, fact illustrated by the graphs below.

**Figure 2:**
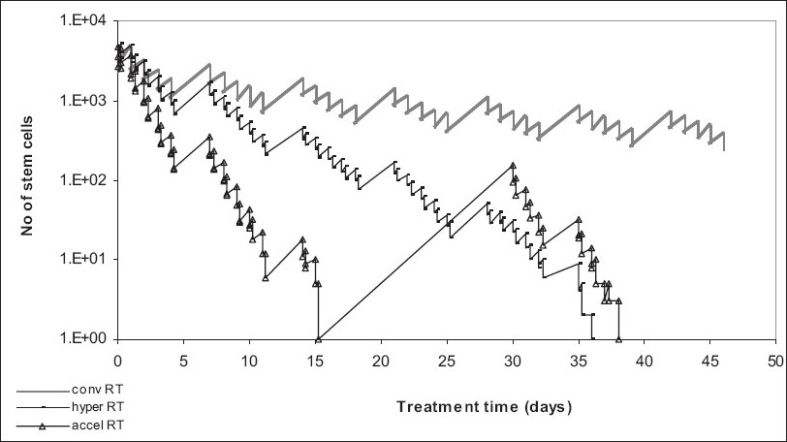
Conventional versus altered fractionated radiotherapy in head and neck cancer

Figure 2 presents three cell survival curves (number of stem cells as a function of treatment time) each illustrating one treatment regimen: conventional, hyperfractionated (1.2 Gy/fraction twice a day, five days per week, over seven weeks) and accelerated radiotherapy (RTOG trial: 1.6 Gy/fraction twice a day, total dose of 67.2 Gy over six weeks with two weeks break after 38.4 Gy), respectively. The graphs [Figure 2] show a clear benefit (tumor kill) from the altered fractionation schedules and a poor tumor control when conventional treatment is employed. Hyperfractionation presents a slight advantage over accelerated fractionation which might be due to the interruption of treatment (or the non-optimal timing of the interruption) because of normal tissue sparing.

To evaluate the effectiveness of various altered fractionation schedules, the main schedule-related parameters (dose per fraction, interfraction interval and the length of treatment gap) have been included in the model and the results presented:

### Influence of Dose Per Fraction on Tumor Control

**Figure 3:**
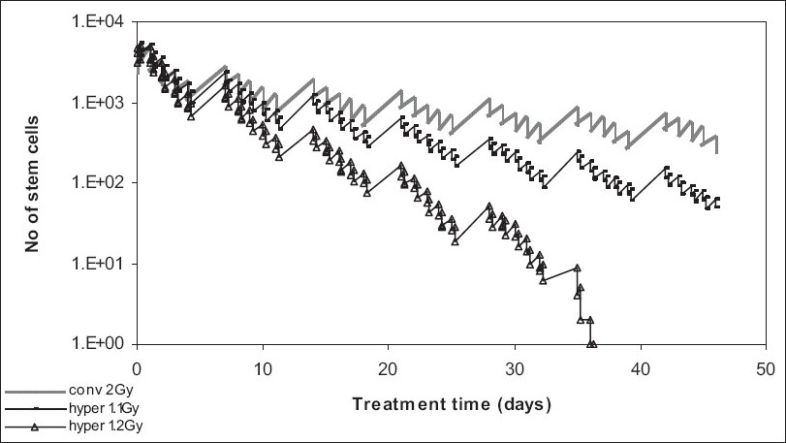
Effect of dose/fraction on tumor control

Figure 3 represents the results of treatment simulation with conventionally fractionated radiotherapy (2 Gy) and also with two hyperfractionated schedules, with 1.1 Gy twice a day and 1.2 Gy twice a day, respectively, over the same period of time (seven weeks) [Table 1].

Again, conventionally fractionated treatment is by far the worst treatment choice, as it cannot control the stem cell population. When it comes to hyperfractionation, the 1.2 Gy/fraction schedule is more efficient than the 1.1 Gy/fraction as it gives a better tumor control (also due to overall higher dose: 84 Gy versus 77 Gy).

### Influence of Interfraction Interval on Tumor Control

**Figure 4:**
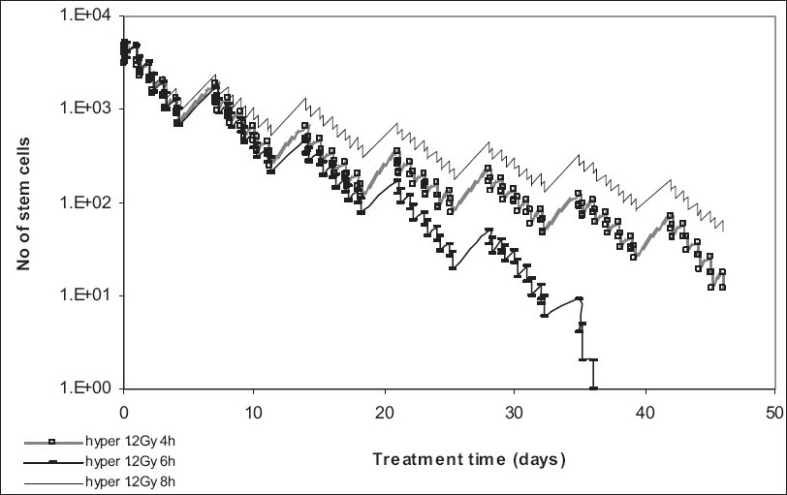
Effect of interfraction interval on tumor control

The time interval between fractions can also have a great impact on tumor control [Table 2]. While the eight-hour interval might be safer for the normal tissue, the six-hour gap leads to a higher tumor control which is because of the interplay between the mechanisms of repopulation and the killing effect of radiotherapy, meaning that the eight-hour time interval is long enough for the tumor to start re populating due to the accelerated stem cell division mechanism [Figure 4].

**Table 2:** Variation of time interval between fractions

*Treatment schedules:*
1.2 Gy twice a day, 4 h interfraction interval, 5 days a week, over 7 weeks, 84 Gy overall dose 1.2 Gy twice a day, 6 h interfraction interval, 5 days a week, over 7 weeks, 84 Gy overall dose 1.2 Gy twice a day, 8 h interfraction interval, 5 days a week, over 7 weeks, 84 Gy overall dose

**Figure 5:**
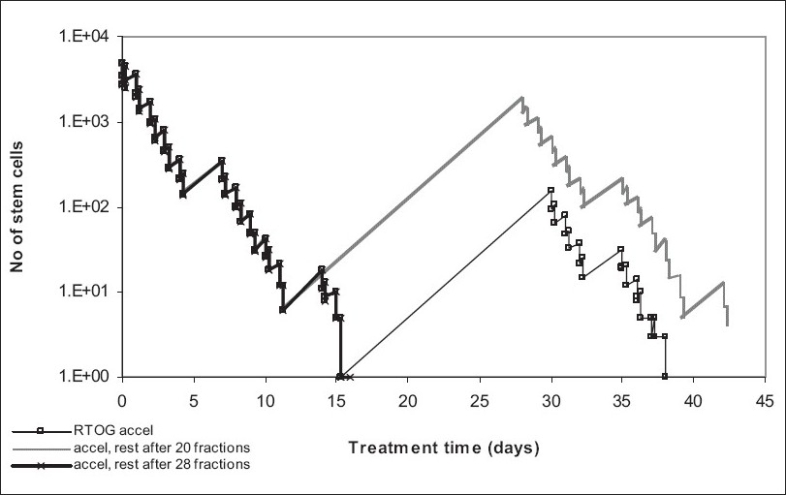
Effect of interfraction interval on tumor control

Further, the four-hour time interval, though not ideal for tumor control either, might be too short to allow normal tissue recovery. The results of this model are in agreement with the RTOG 83-13 report[13] which has concluded that the interfraction interval is a significant independent factor for the development of grade 3 or 4 late effects. Fu *et al*[11] have shown that patients who received hyperfractionated radiotherapy with an interval of less than or equal to 4.5 hours had significantly more grade 3+ late effects than those who received the two daily consecutive fractions at least 4.5 hours apart. Therefore, the interfraction interval employed by the RTOG trial was six hours.[11]

### Influence of Treatment Gap on Tumor Control

Accelerated repopulation, one of the main reasons for local failure of head and neck squamous cell carcinoma after radiotherapy, is clearly illustrated in [figure 5], where various treatment gaps during accelerated therapy have allowed the surviving stem cells to thrive and repopulate more aggressively than before the start of treatment [Table 3].

**Table 3:** Variation of treatment gap timing

*Treatment schedules:*
1.6 Gy twice a day, over 6 weeks, total dose of 67.2 Gy, with 2 weeks break after 38.4 Gy (after 24 fractions) (RTOG trial) 1.6 Gy twice a day, over 6 weeks, total dose of 67.2 Gy, with 2 weeks break after 32 Gy (after 20 fractions) 1.6 Gy twice a day, over 6 weeks, total dose of 67.2 Gy, with 2 weeks break after 44.8 Gy (after 28 fractions)

The treatment gap employed by the RTOG trial (after 24 fractions), as shown by the graph, is the most plausible out of the three schedules presented. When timing the gap only after 20 fractions, the stem population is still large enough to rebuild the tumor by accelerated repopulation. A treatment gap scheduled after 28 fractions, though successful for the tumor, might be too aggressive for the normal tissue.

The model shows that scheduling treatment gaps during radiotherapy should be done cautiously as instead of overcoming repopulation (which is the aim of accelerated treatment) this process might be encouraged through a wrongly timed treatment break. Although accelerated radiotherapy is still a powerful regimen in overcoming tumor repopulation, the treatment gap, if any, has to be planned with tumor kinetics in mind, especially for highly proliferating tumor like squamous cell carcinoma of the head and neck.

Several trials have been comparing the benefits of the altered fractionation schedules on advanced head and neck cancers. The general conclusion, resulted from the meta analysis published by Bourhis *et al,*[14] is that “altered fractionated radiotherapy improves survival in patients with head and neck squamous cell carcinoma. Comparison of the different types of altered radiotherapy suggests that hyperfractionation has the greatest benefit”. The results of a phase III randomized trial have shown a 24% increase in loco-regional control (maintained at five years) when an accelerated treatment regimen (62 to 64 Gy in 31 to 32 fractions over 22 to 23 days (2 Gy/fraction twice daily)) was compared to a conventional one (70 Gy to the primary tumor over seven weeks and 35 fractions of 2 Gy in 49 days).[14]

## Conclusions

Head and neck cancer can, without doubt, benefit from altered fractionation schedules as it has been proven that altered fractionation offers superior tumor control to conventional radiotherapy. While hyperfractionation might offer advantages over accelerated radiotherapy regarding tumor control, the correct selection of schedule-related parameters for both treatment types is crucial in overcoming accelerated repopulation. Therefore, the treatment-related parameters leading to effective altered fractionation schedules, as indicated above and also confirmed by successful trials, are: 1.2 Gy/fraction twice a day, with a six-hour interfraction interval, over seven weeks. Nevertheless, additional treatment modifiers are needed to further improve the prognosis of head and neck cancer patients.

Modeling of treatment regimens and their input parameters can offer a comprehensive understanding of the radiobiological interactions and also the treatment outcome. Therefore, models are needed to open further research avenues, to suggest relationships between radiobiological parameters, and lead us towards the optimum treatment schedule for today's most fought disease.
